# Critical Role of Gap Junction Coupled K_ATP_ Channel Activity for Regulated Insulin Secretion

**DOI:** 10.1371/journal.pbio.0040026

**Published:** 2006-01-17

**Authors:** Jonathan V Rocheleau, Maria S Remedi, Butch Granada, W. Steven Head, Joseph C Koster, Colin G Nichols, David W Piston

**Affiliations:** **1**Department of Molecular Physiology and Biophysics, Vanderbilt University Medical Center, Nashville, Tennessee, United States of America; **2**Department of Cell Biology and Physiology, Washington University School of Medicine, St. Louis, Missouri, United States of America; Stanford UniversityUnited States of America

## Abstract

Pancreatic β-cells secrete insulin in response to closure of ATP-sensitive K^+^ (K_ATP_) channels, which causes membrane depolarization and a concomitant rise in intracellular Ca^2+^ (Ca_i_). In intact islets, β-cells are coupled by gap junctions, which are proposed to synchronize electrical activity and Ca_i_ oscillations after exposure to stimulatory glucose (>7 mM). To determine the significance of this coupling in regulating insulin secretion, we examined islets and β-cells from transgenic mice that express zero functional K_ATP_ channels in approximately 70% of their β-cells, but normal K_ATP_ channel density in the remainder. We found that K_ATP_ channel activity from approximately 30% of the β-cells is sufficient to maintain strong glucose dependence of metabolism, Ca_i_, membrane potential, and insulin secretion from intact islets, but that glucose dependence is lost in isolated transgenic cells. Further, inhibition of gap junctions caused loss of glucose sensitivity specifically in transgenic islets. These data demonstrate a critical role of gap junctional coupling of K_ATP_ channel activity in control of membrane potential across the islet. Control via coupling lessens the effects of cell–cell variation and provides resistance to defects in excitability that would otherwise lead to a profound diabetic state, such as occurs in persistent neonatal diabetes mellitus.

## Abstract 2

Gap junctions electrically couple pancreatic β-cells, and can preserve excitability (glucose sensitivity and insulin secretion) in the islet even when a majority of cells have nonfunctional ATP-activated potassium channels.

## Introduction

β-cells within the intact islet of Langerhans exhibit synchronous glucose-dependent bursts of electrical activity. Dissociated β-cells also show increased electrical activity at elevated glucose concentrations, but this activity is variable from cell to cell, with some cells being electrically “silent” and others continuously “bursting” at any given glucose concentration [1–4]. Synchronization of electrical activity via gap junctions has long been argued as essential for normal glucose-dependent insulin secretion in the intact islet [5–8]. In particular, islets devoid of gap junction–forming Connexin 36 (Cx36) exhibit limited synchronization of glucose-stimulated intracellular Ca^2+^ (Ca_i_) [8]. To test the extent and role of this coupling in the islet electrical response, it would be desirable to examine within the intact islet the behavior of cells that would be silent or bursting as isolated cells. To date, it has not been possible to know which cell is which in the setting of the intact islet. However, we have generated transgenic mice containing two electrically distinct types of β-cells that uniquely permit us to track the behavior of specific individual cells within the intact islet [9]. As previously shown, these mice express a β-cell–specific, dominant-negative Kir6.2[AAA]-GFP transgene, in which the pore-forming subunit of the ATP-sensitive K^+^ (K_ATP_) channel is rendered nonfunctional. Mosaic expression of the Kir6.2[AAA]-GFP transgene results in a high-level expression in approximately 70% of the β-cells, and these expressing cells are distributed randomly throughout the islet [9,10]. Thus, K_ATP_ channels are functionally “knocked out” of 70% of cells, yet the intact islets still show glucose-dependent insulin secretion, with an even steeper concentration dependence of insulin secretion than wild-type (WT) islets [9].

## Results/Discussion

**Figure 1:**
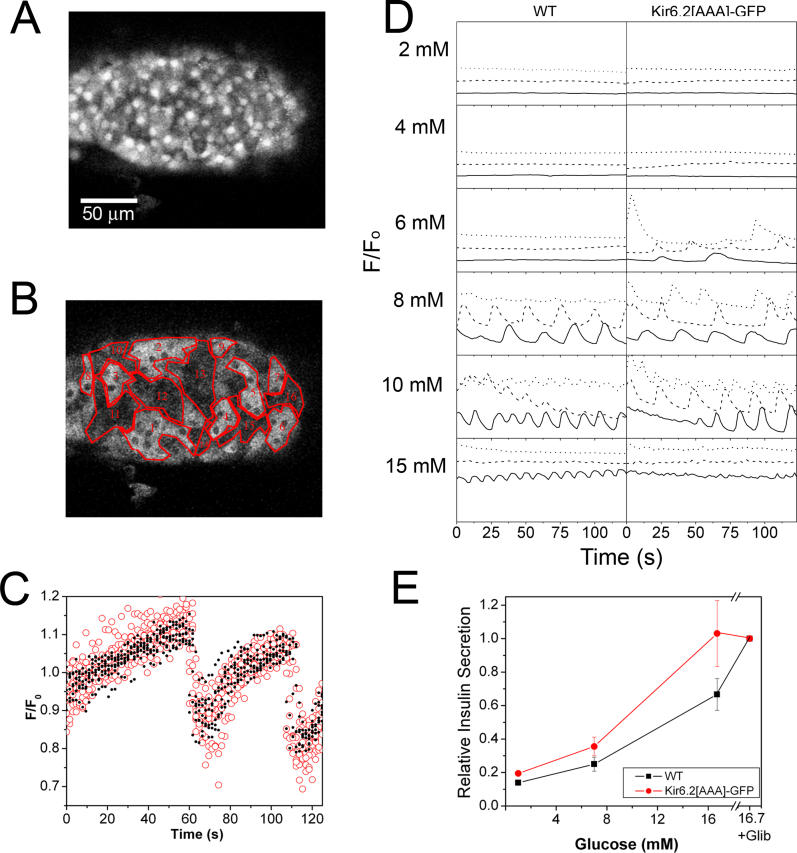
Whole-Islet Ca_i_ and Insulin Responses (A) Fluorescence (488-nm excitation and 620–680-nm emission band-pass filter) image of a Fura Red–labeled Kir6.2[AAA] islet. As with labeling of intact WT islets, the fluorescence intensity of Kir6.2[AAA] islets shows increased intensity in the nucleus and relatively little intensity variance across the tissue. (B) Fluorescence (488-nm excitation and a 540/20-nm band-pass filter) image of the same islet. Note that in (A) the islet is uniformly labeled and there is no indication of GFP fluorescence bleed-through. In (B), 16 regions that were either positive (eight regions) or negative (eight regions) for Kir6.2[AAA]-GFP transgene expression, are outlined in red. (C) The Ca_i_ responses in 6 mM glucose from groups of cells (outlined in [B]) that are either Kir6.2[AAA]-positive (filled black circle) or -negative (open red circle) demonstrated identical oscillatory behaviors, indicating that both are in synchrony. (D) A comparison of glucose-stimulated Ca_i_ responses measured using Fluo-4 from WT (left) and Kir6.2[AAA] (right) transgenic islets. Islets were exposed to the indicated glucose concentrations for greater than 10 min by changing the reagent well solution. Images were then collected at 1.5-s intervals over a period of 125 s. In contrast to the traces found in (C), each trace in each panel (solid, dot, and dashed) represents a *whole single islet*. These islet traces are done to show the dose -response of individual islets rather than synchrony across the tissue. WT islets showed no oscillatory behavior until treated to 8 mM glucose or greater (responses of three different islets are shown). Kir6.2[AAA] islets were also quiescent at low glucose concentrations; however, these islets showed Ca_i_ oscillations at 6 mM glucose or above (*n* = 5 islets). (E) Glucose-stimulated insulin secretion from WT and Kir6.2[AAA] islets (mean ± SEM, *n* = 17 and 15, respectively). Islets were also exposed to 16.7 mM glucose + glibenclamide (Glib) to achieve maximum possible insulin secretion. The difference in means ± 95% confidence interval is shown in Figure S2A.

**Figure 2:**
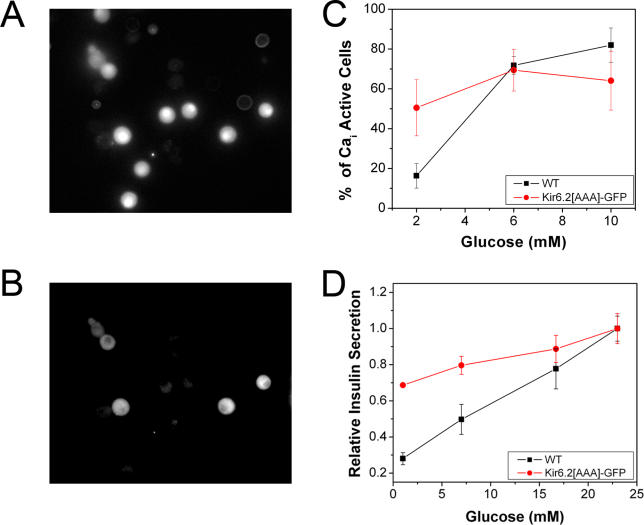
Dispersed Cell Ca_i_ and Insulin Responses (A) Dispersed Kir6.2[AAA] cells labeled with Fura-2 (340-nm image). These are mainly β-cells, with the expected minority of non–β-cells. (B) GFP image of the same field of view of (E). A fraction of cells (four of eight) are GFP positive. (C) The glucose-stimulated Ca_i_ response of dispersed cells from WT (black square) and Kir6.2[AAA] (red circle) islets measured using Fura-2 fluorescence microscopy. Kir6.2[AAA] cell preparations were a combination of GFP-labeled and non-labeled cells. The percentage of Ca_i_-active cells was measured in 2, 6, and 10 mM glucose on three separate days in WT and Kir6.2[AAA] dispersed cells, respectively (mean ± standard error of the mean [SEM], *n* = 3). The WT cells show a strong glucose dependence, whereas the Kir6.2[AAA] cells show similar numbers of Ca_i_ active cells at all glucose concentrations. (D) Insulin secretion from dispersed WT and Kir6.2[AAA] cells (mean ± SEM, *n* = 4) from static 1-h incubations. This incubation time shows K_ATP_ channel–dependent insulin secretion, but does not encompass K_ATP_ channel–independent insulin secretion shown to occur in knockout islets beyond 3 h of incubation [13]. The difference in means ± 95% confidence interval for (C) and (D) are shown in Figure S2B and S2C, respectively).

To examine the behavior of individual cells within the intact islet, we developed a microfluidic device to hold pancreatic islets stationary while under continuous fluid flow (see Figure S1) [11]. We compared the glucose-dependent Ca_i_ responses of WT and Kir6.2[AAA] transgenic islets (Figure 1). Near the periphery of the islet, fluorescence from a Ca^2+^ sensor (Fura Red) was relatively uniform in all cells (Figure 1A), and spectrally separable from green fluorescent protein (GFP) fluorescence (Figure 1B). Both the GFP-positive and -negative populations of β-cells (K_ATP_-absent and K_ATP_-present cells, respectively) within a Kir6.2[AAA] transgenic islet showed identical Ca_i_-responses (Figure 1C). WT islets were “Ca_i_ inactive” across the tissue below 8 mM glucose (i.e., Ca_i_ remained low and no oscillations were observed; Figure 1D), consistent with previous studies [9,11]. Kir6.2[AAA] islets were also completely Ca_i_ inactive across the tissue at low glucose concentrations (2 and 4 mM), but they exhibited Ca_i_ activity at slightly lower glucose levels than WT (6 mM). This leftward shift in glucose dependence of Ca_i_ in Kir6.2[AAA] islets is consistent with the leftward shift in whole-islet glucose-dependent insulin secretion (Figure 1E) [9]. Kir6.2^−/−^ and SUR1^−/−^ mice, which completely lack K_ATP_ channels, show essentially no glucose dependence of electrical activity, Ca_i_, or insulin secretion [12–14]. Thus, the near-normal glucose response of Ca_i_ (Figure 1D) and insulin secretion (Figure 1E) by Kir6.2[AAA] islets are initially somewhat surprising, since K_ATP_ is functionally knocked out in approximately 70% of the β-cells. Paracrine signaling could influence the electrical synchronization between islet cells, for instance by δ-cell secretion of somatostatin [15]. However, these data suggest that K_ATP_ channel activity in the residual 30% of cells is coupled through gap junction conductance that is strong enough to hyperpolarize the knockout cells. This suggestion, in turn, predicts that glucose dependence will be lost in dispersed Kir6.2[AAA]-GFP cells. Accordingly, we measured the glucose-stimulated Ca_i_ response and insulin secretion from dispersed cell preparations (Figure 2). Image fields of isolated Kir6.2[AAA] β-cells show uniform loading of the Ca^2+^ indicator dye (Fura-2; Figure 2A), and no overlap with GFP fluorescence (Figure 2B). Both the Ca_i_ response and insulin secretion were essentially glucose independent in dispersed Kir6.2[AAA] cells (Figure 2C and 2D). This is in striking contrast to the Ca_i_ response and insulin secretion from dispersed WT cells which are strongly glucose dependent, but is consistent with previous results from SUR knockout mice [16].

**Figure 3:**
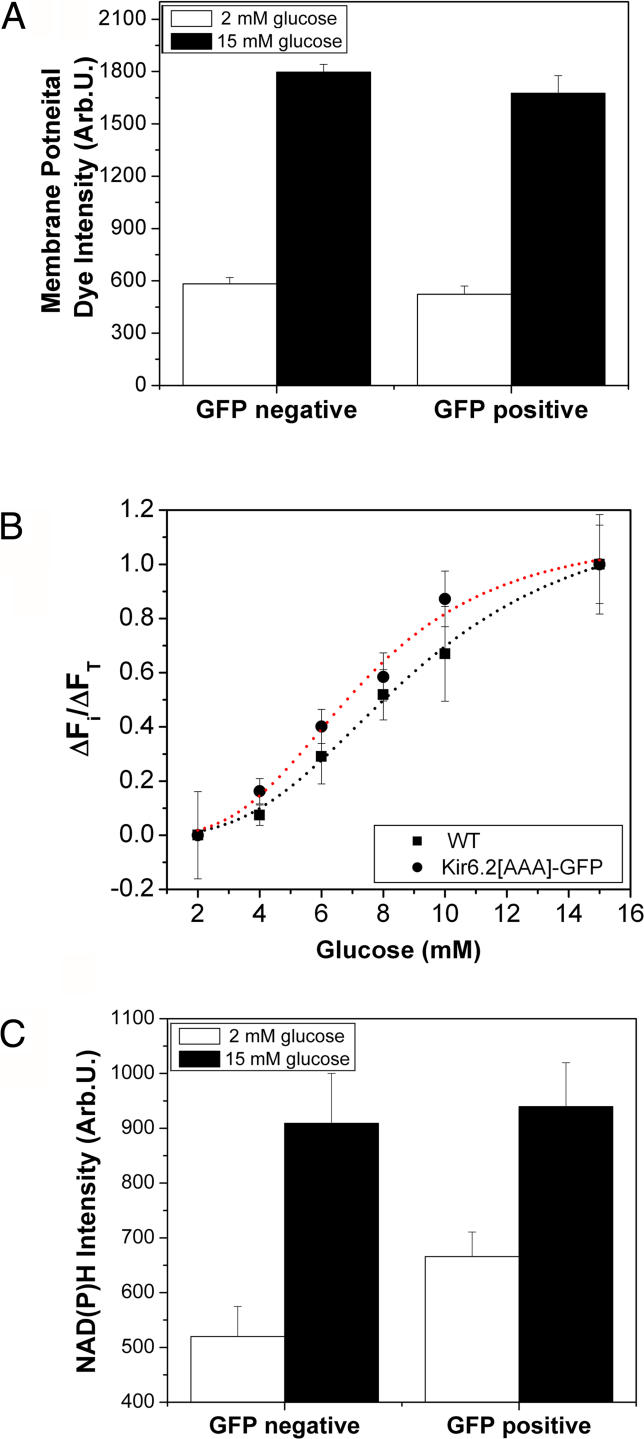
Membrane Potential and Metabolic Response of Kir6.2[AAA] Islets and Cells (A) The membrane potential of GFP-positive and -negative cells within a Kir6.2[AAA] islet. Islets were bathed in 500 nM of membrane potential dye (DiSBAC_2_(3)) (*n* = 4). This oxanol dye enters cells in a membrane potential–dependent manner with increasing intensity as a cell becomes depolarized, and is spectrally distinct from GFP. The GFP-positive and -negative cells showed similar intensity (membrane potential) at 2 and 15 mM glucose stimulation, and had responses consistent with the responses observed in WT islets (data not shown). (B) The NAD(P)H glucose-dose response of WT (*n* = 9) and Kir6.2[AAA]-GFP (*n* = 10) islets. Device-trapped islets were treated to the indicated glucose concentrations for more than 5 min prior to image collection. The normalized change in NAD(P)H fluorescence intensity (ΔF/ΔF_T_) is plotted versus glucose stimulation. The black and red dotted lines are the data fitted to a Hill equation with Km values of 9.0 ± 0.7 and 7.6 ± 0.7 mM for the WT and Kir6.2[AAA] islets, respectively. (C) The NAD(P)H responses of dispersed GFP-negative and -positive Kir6.2[AAA] cells. The cells were incubated in 2 mM glucose (open bars) prior to incubation for 5–10 min with 15 mM glucose (closed bars). GFP fluorescence was used for the distinction as GFP negative or positive. NAD(P)H-intensity measures were taken from cells in four fields of view from nine separate dishes of Kir6.2[AAA] dispersed cells (*n* = 9).

It was previously shown that dispersed GFP-positive Kir6.2[AAA] β-cells are continuously depolarized in nonstimulatory glucose concentrations [9]. To examine whether cell–cell coupling reconstitutes glucose-dependent membrane potentials in intact islets, we imaged intact islets using a membrane potential–sensitive dye (Figure 3A). At 2 mM glucose, a few cells on the islet periphery were noticeably more depolarized than others. These bright cells were never seen to co-localize with GFP fluorescence (not shown), and were likely α-cells. The rest of the islet cells showed relatively dim uniform fluorescence, consistent with uniformly polarized membranes, and all of these cells brightened (depolarized) upon glucose stimulation. GFP-expressing and non-expressing cells had indistinguishable membrane polarization (Figure 3A). Thus, β-cells across the Kir6.2[AAA] islet have similar resting membrane polarization, and all depolarize with increased glucose concentrations.

In contrast to electrical activity, we have not observed metabolic coupling of β-cells [11,17], although there may be feedback between electrical activity and metabolic response. The Kir6.2[AAA] islets provide a unique opportunity to examine this potential feedback. We used intrinsic NAD(P)H fluorescence as a measure of cellular metabolic status [11,18]. In comparison to WT, Kir6.2[AAA] islets showed no difference in baseline NAD(P)H intensity, but showed a slight (∼1 mM) leftward shift in the (glucose) response (Figure 3B). This shift is comparable to that of glucose-stimulated Ca_i_ response and insulin secretion (see Figure 1D and 1E), suggesting a feedback modulation of metabolism during electrical activity. Such feedback could result from Ca_i_ influx [19] or insulin signaling [20,21] since both likely modulate β-cell metabolism and occur when the tissue is electrically active. We further compared the baseline and stimulated responses of dispersed Kir6.2[AAA] cells (Figure 3C). Baseline NAD(P)H intensity was significantly elevated in GFP-positive cells (1.3-fold vs. GFP negative at 2 mM glucose), but was similar upon glucose stimulation. The elevated basal metabolism in GFP-positive cells with no difference in glucose-stimulated metabolism is again consistent with a modulating effect of Ca_i_ or insulin**.** Thus, even though metabolism is not directly coupled between cells in the islet [11,17], entrained electrical activity and shifted Ca_i_ responses can influence metabolic response.

**Figure 4:**
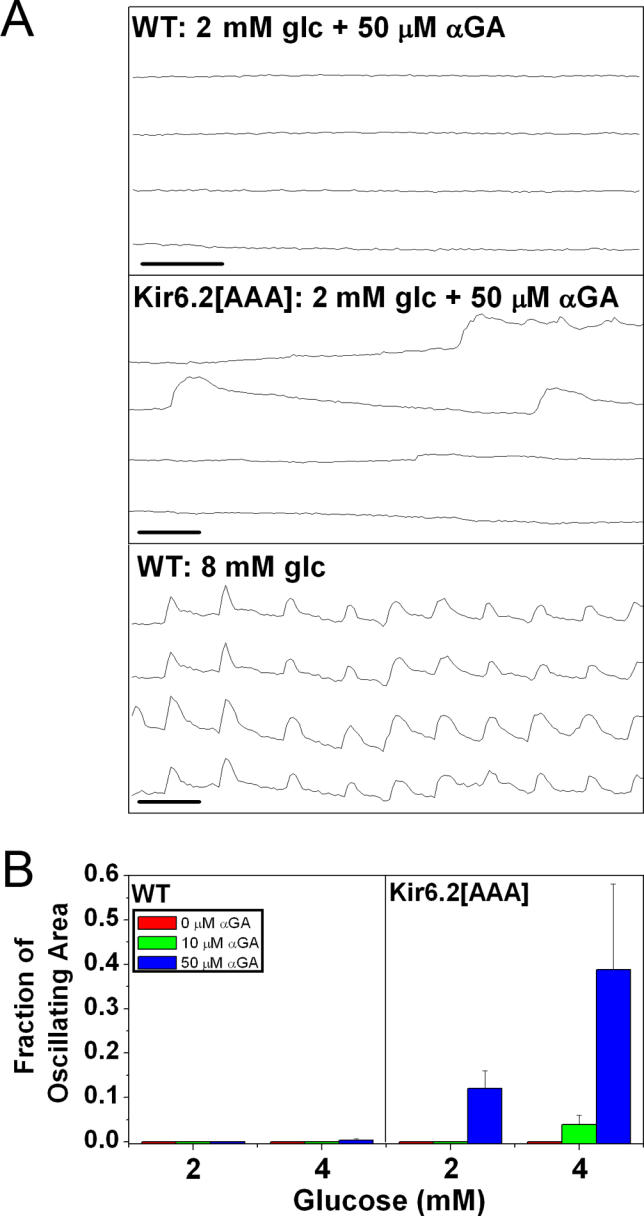
The Ca_i_ Responses of WT and Kir6.2[AAA] Islets in the Presence of αGA, a Gap Junction Inhibitor (A) Fluo-4, AM–labeled WT and Kir6.2[AAA] islets in 2 mM (*n* = 4 and 5, respectively) or 4 mM (*n* = 5 and 6, respectively) glucose (glc) were exposed to 0, 10, or 50 μM αGA. The islets were imaged to provide relative Ca_i_ traces from the Fluo-4 response, and each panel shows the response in four different regions of a single islet. The representative traces shown are from a WT islet in 2 mM glucose with 50 μM αGA (WT: 2 mM glc + 50 μM αGA, *n* = 4), a Kir6.2[AAA] islet in 2mM glucose with 50 μM αGA (Kir6.2[AAA]: 2 mM glc + 50 μM αGA, *n* = 5), and a WT islet in 8 mM glucose (WT: 8 mM glc). The black bar in each panel indicates 30 s. (B) The oscillating (Ca_i_ active) area and total islet area were measured in each islet under each condition. The data are expressed as the fraction of oscillating area, calculated as the oscillating area divided by the total area (mean ± SEM) from WT islets in 2mM (*n* = 4) and 4 mM glucose (*n* = 5), and Kir6.2[AAA] islets in 2 mM (*n* = 5) and 4 mM glucose (*n* = 6).

The above results suggest a model whereby the Kir6.2[AAA] islet response is due to coupling of K_ATP_ channel activity, presumably through gap junctions. This model predicts that inhibition of gap junctions would recapitulate the uncoupled Ca_i_ activity found in dispersed Kir6.2[AAA] cells. Thus, we examined glucose-stimulated Ca_i_ responses in the presence of a gap junction inhibitor, 18α-glycyrrhetinic acid (αGA) [22]. Ca_i_ measurements of WT islets (Figure 4A) showed no β-cell oscillations at sub-threshold glucose levels in the presence or absence of either 10 or 50 μM αGA. In contrast, spontaneous Ca_i_ oscillations were observed in the presence of 50 μM αGA in four of five Kir6.2[AAA] islets at 2 mM glucose, and in six of six Kir6.2[AAA] islets at 4 mM glucose (Figure 4B, Videos S1–S3). Unlike the synchronous oscillations across the entire islet that are seen in the absence of αGA (Figure 4A, bottom trace, and Figure 1C), these oscillations occurred asynchronously in small groupings of cells (Figure 4A, middle trace). The resulting asynchronous Ca_i_ activity in portions of Kir6.2[AAA] islets is consistent with partial decoupling of the K_ATP_ channel activity by the inhibition of gap junction conductance.

**Figure 5:**
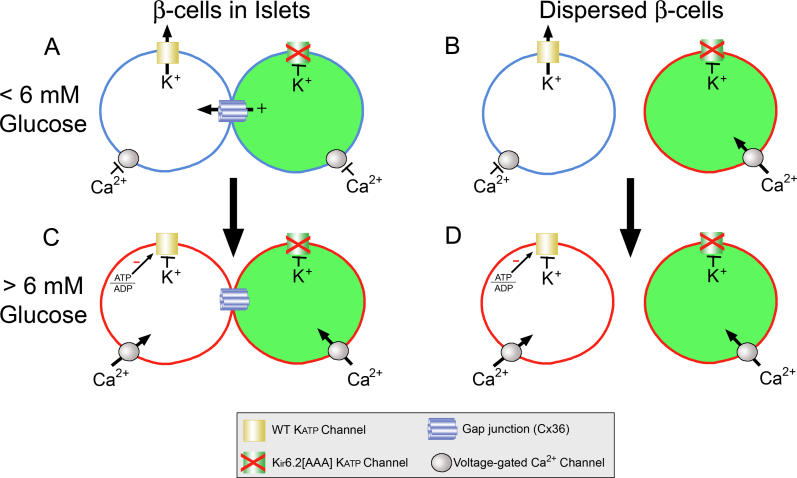
Schematic Model of the Responses of β-cells in the Islet and Dispersed β-cells from Kir6.2[AAA] Transgenic Mice in Both Low (<6 mM) and High (>6 mM) Glucose Concentrations (A) and (C) show the β-cell responses in the islet, and (B) and (D) show the dispersed β-cell responses. (A) A WT β-cell has normal K_ATP_ channel activity (white cell), which maintains plasma membrane potential (blue membrane). The GFP-tagged Kir6.2[AAA] cell (green cell) would normally be depolarized; however, the K_ATP_ current from the normal cell is coupled through gap junctions to maintain membrane polarity (blue membrane). (B) In the absence of coupling through gap junctions, the cell with normal K_ATP_ channel activity (white cell) maintains polarity (blue membrane), but the Kir6.2[AAA] cell depolarizes (red membrane) resulting in Ca_i_ influx through voltage-gated channels. (C) The addition of glucose raises the ATP/ADP ratio, which closes the K_ATP_ channel in the normal cell. The loss of this K^+^ current results in membrane depolarization (red membrane) in both cells, and Ca_i_ influx through voltage-gated Ca^2+^ channels. (D) In the dispersed cells, this rise in ATP/ADP ratio results in depolarization and Ca_i_ influx occurring in both cells.

The data presented here indicate that glucose control of membrane potential, Ca_i_, and insulin secretion all depend critically on the gap junctional coupling of K_ATP_ channel activity across neighboring cells within the islet (Figure 5). It has previously been shown that knockout of Cx36 in islets results in reduction of glucose-stimulated electrical synchrony [8]. Our results provide additional experimental evidence for gap junctional coupling between islet β-cells. Furthermore, our results demonstrate the mechanism by which the coupling of K_ATP_ channel activity via gap junctions results in near-normal glucose-dependent electrical and Ca_i_ responses in the intact islet, even when a majority of β-cells are essentially glucose insensitive. More specifically, this coupling allows β-cells in low glucose with normal K_ATP_ channel activity to clamp the membrane potential of neighboring cells. The electrical properties of isolated β-cells are very variable [2–4]. This variability along with our results suggests that the rise in basal insulin secretion from Cx36 knockout islets is due to an inability of quiescent cells to clamp the membrane potential of other β-cells with low stimulation threshold. This may also explain an evolutionary drive to organize these secretory cells into a unique electrical syncytium. Such a mechanism provides a protective effect against hypoglycemia if individual cell properties change under pathological or physiological stimuli. Factors that reduce K_ATP_ channel density, such as channel subunit trafficking mutants [23,24], could do so to the point where individual cells become glucose insensitive. However, the remaining glucose-sensitive cells in the islet with sufficient residual K_ATP_ channel activity will maintain a glucose response of the whole islet. Such cell coupling would help resist the development of persistent hypoglycemic hyperinsulinemia (PHHI), unless compounded by additional defects, as may be the case in human PHHI [25].

Our results also have implications for the understanding of diabetic phenotypes resulting from K_ATP_ channel gain of function or from other causes of hypoexcitability. It is now clear that overactive K_ATP_ channels cause neonatal diabetes due to inexcitability of β-cells and failure to secrete insulin [26,27]. Our data demonstrate that cell–cell coupling can ensure maintained hyperpolarization in all cells if K_ATP_ channel activity in just a few reaches a threshold of inexcitability. However, this also suggests that the domination of K_ATP_ channel strength on neighboring cell membrane potentials, may permit elevated K_ATP_ channel activity in only a few β-cells to clamp the remainder at a hyperpolarized potential, thereby stopping secretion. Such a mechanism may underlie the profound diabetic state in persistent neonatal diabetes mellitus [27]. Equivalent gain-of-function model systems [26] and the experimental approach that we describe here should allow a critical test of the syncytial involvement in these types of neonatal diabetes.

## Materials and Methods

### Islet isolation and secretion measurements

Islets were isolated as previously described [28,29] and maintained in complete RPMI medium 1640 containing 10% fetal bovine serum and 11 mM glucose at 37 °C under 5% humidified CO_2_ for 24–72 h. Islets were dispersed to single cells as described previously [9]. Insulin release was measured from pancreatic islets (ten per well) or dispersed cells (equivalent to 15 islets per well) in static 1-h incubations as described previously [9].

### Confocal imaging of microfluidic device trapped islets

Devices were fabricated using the elastomer polydimethylsiloxane (PDMS) as described elsewhere [30] (see Figure S1 for details of fabrication and use). Islets were labeled with 4 μM of either Fluo-4, acetoxymethyl (AM) or Fura Red, AM at room temperature for 1 to 2 h in imaging buffer (125mM NaCl, 5.7 mM KCl, 2.5 mM CaCl_2_, 1.2 mM MgCl_2_, 10 mM HEPES, 2 mM glucose, and 0.1% bovine serum albumin [pH 7.4]). One- and two-photon microscopy was performed on a LSM 510 microscope with a 20 × 0.75 NA Fluar objective lens (Carl Zeiss, Thornwood, NewYork, United States). The device was held on the microscope in a humidified temperature controlled stage (Carl Zeiss) for imaging at 37 °C. Fluo-4 (Molecular Probes, Eugene, Oregon, United States) was imaged using the 488-nm laser line and the long-pass 505-nm emission filter. NAD(P)H intensity was imaged with a 710-nm mode-locked Ti:Saph laser (∼3.5 mW at the sample) and fluorescence collection through a non-descanned detector with a custom 380- to 550-nm filter (Chroma, Rockingham, Vermont, United States) [18]. We collected four focal planes separated by 3-μm intervals. Fura Red (Molecular Probes) was imaged using the 488-nm laser line and a band-pass filter (620–680 nm). The GFP label of Kir6.2[AAA] islets was imaged using the 488-nm laser and band-pass (540/20) filter, with no noticeable bleed-through of the Fura Red signal. Membrane potential was measured with bis-(1,3-diethylthiobarbituric acid)trimethine oxonol (DiSBAC_2_(3); Molecular Probes). This dye accumulates in the cells in a Nernst-dependent manner [31]. We flowed the indicated solutions with 500 nM of DiSBAC_2_(3). These solutions were allowed to equilibrate for approximately 30 min to allow dye penetration before imaging. DiSBAC_2_(3) images were collected by 543-nm excitation and a long-pass 560-nm filter. GFP fluorescence in these same islets was imaged separately using 488-nm excitation and a 500–530-nm band-pass filter.

### Single cell Ca_i_ measurements

Prior to imaging, cells were labeled with 2 μM Fura-2, AM (Molecular Probes) for 30 min at room temperature and washed with imaging buffer containing 2 mM glucose. Imaging was done on a TE300 Eclipse microscope (Nikon, Melville, New York, United States) using the 40 × 1.3 NA DIC PlanFluor lens, side-mounted CoolSnap HQ camera (Roper Scientific, Tucson, Arizona, United States), and Fura-2 filter set (Chroma). The cells were placed on a stage enclosed in Plexiglas and kept in humidified air at 37 °C. The system was controlled with Metamorph 6.0 (Universal Imaging, Downington, Pennsylvania, United States). Image pairs were collected at 2-s intervals for 2 min after incubation for at least 10 min in 2, 6, and 10 mM glucose.

### Image analysis

Images were analyzed with MetaMorph 5.0 (Universal Imaging). For Fluo-4 analysis, islets/regions of islets were outlined and their intensities versus time were plotted. From these plots, the relative intensity change (F/F_0_) and frequencies were calculated. For Fura-2 analysis, individual cells were outlined for calculation of the background corrected 340:380-nm intensity ratio. Cells were categorized as “calcium active” if they had a ratio that was either more than two standard deviations larger than that observed for WT cells in 2 mM glucose or demonstrated a change of more than one standard deviation change during the 2-min observation period.

## Supporting Information

Figure S1 A Microfluidic Device, Designed to Hold a Pancreatic Islet Stationary in a Fluid Stream, was Fabricated Using the Elastomer Polydimethylsiloxane (PDMS) The fabrication of the device is described in [30].
(A) A dye-loaded microfluidic device. The image is labeled to show the Reagent well, Islet In/Out port, Wall trap area, and Waste port. This device relies on gravity flow from either the Reagent well or Islet In/Out port to the Waste port. Islets were brought into the device through the In/Out port. The islet travels through the main channel of the device until it comes in contact with Wall area.
(B) A differential interference contrast image of a pancreatic islet at the Wall trap area of the microfluidic device. This islet is in the main channel (height ∼100 μm and width ∼600 μm) touching the wall trap (bottom of image), with fluid flowing by gravity from the top to bottom of the image. Islets trapped in a microfluidic device channel with approximately 100 μm height are pressed against the coverslip surface for optimal microscopic imaging. The islet is held stationary by the coverslip, ceiling and wall trap. All the islets studied maintain their shape throughout the experiments, which suggests limited sheer pressure. We have previously shown that WT islet Ca_i_ and NAD(P)H responses are unperturbed in similar devices [11].
(C) Multiple image planes of sulforhodamine B (0.2 μM) dye as it is flown past a device-trapped islet. The *z-*distance from the coverslip is shown at the bottom-left corner of each image. Note that the dye solution is not observed in the wall trap area above 16 μm of depth. In this region of the device, the height of the channel drops from approximately 100 to 15 μm. This channel height allows fluid to flow while blocking islet movement. Once islets are trapped in the device, we plugged the In/Out port and started gravity flow from the Reagent well.
(D) Changing the Reagent well solution (100 μl) to contain sulforhodamine B (0.2 μM) resulted in complete change of solution at >1 min. The half-maximum of this change was 27 s, which corresponds to a flow rate of approximately 1.2 μl min^−1^, which is below the calculated maximum velocity (∼3 μl min^−1^), but slightly faster than the calculated flow rate (∼0.7 μl min^−1^). At this flow rate, we observed no noticeable warping of the islet, which is consistent with low sheer. Furthermore, the islets remained stationary after long periods of flow time (minutes to hours) and during reagent solution changes. Keeping the islet in such a stationary position facilitates time-lapse imaging and the observation of similar regions after many different treatments.
(4 MB TIF).

Figure S2 Difference in Means with Confidence Intervals for Figures 1E, 2C, and 2D Experimental details are as described in the text and figure captions. The difference in means (X¯_1_−X¯_2_) for each data point is plotted ± 95% confidence interval with the zero difference shown with a dashed line.
(A) Data from Figure 1E.
(B) Data from Figure 2C.
(C) Data from Figure 2D.
(6.1 MB TIF).

Video S1 This Video Shows a Kir6.2[AAA] Islet Treated to 2 mM Glucose (2 mM Glc) The elapsed time covers a period of 190 s at 1.57 frames/s. Some individual cells are observed to asynchronously increase in intensity. These responses are similar to those observed in WT islets at nonstimulatory glucose concentrations [11], and are consistent with an α-cell response.
(407 KB MOV).

Video S2 This Video Shows the Same Kir6.2[AAA] Islet Shown in Video S1, but Treated to 2 mM Glucose with 10 μM αGA (2 mM Glc + 10 uM GA) The elapsed time covers a period of 190s at 1.57 frames/s. Similar to the response found with 2 mM glucose (Video S1), some individual cells are observed to asynchronously increase in intensity. These responses are similar to those observed in WT islets at nonstimulatory glucose concentrations [11], and are consistent with an α-cell response.
(438 KB MOV).

Video S3 This Video Shows the Same Kir6.2[AAA] Islet Shown in Video S1, but Treated to 2 mM Glucose with 50 μM αGA (2 mM Glc + 50 uM GA) The elapsed time covers a period of 383 s at 1.57 frames/s. White arrows are shown to highlight synchronous Ca_i_ responses among groups of cells within the islet, consistent with a β-cell response. However, these responses occur in two distinct regions of the islet at different time points indicating asynchrony between cell groupings.
(899 KB MOV).

## Abbreviations

αGA: 18α-glycyrrhetinic acid
Ca_i_: intracellular Ca^2+^
Cx36: Connexin 36
GFP: green fluorescent protein
K_ATP_: ATP-sensitive K^+^
PHHI: persistent hypoglycemic hyperinsulinemia
SEM: standard error of the mean
WT: wild-type

## References

[pbio-0040026-b001] Rorsman P, Trube G (1986). Calcium and delayed potassium currents in mouse pancreatic beta-cells under voltage-clamp conditions. J Physiol.

[pbio-0040026-b002] Misler S, Barnett DW, Pressel DM, Gillis KD, Scharp DW (1992). Stimulus-secretion coupling in beta-cells of transplantable human islets of Langerhans. Evidence for a critical role for Ca^2+^ entry. Diabetes.

[pbio-0040026-b003] Atwater I, Rosario L, Rojas E (1983). Properties of the Ca-activated K^+^ channel in pancreatic beta-cells. Cell Calcium.

[pbio-0040026-b004] Zhang M, Goforth P, Bertram R, Sherman A, Satin L (2003). The Ca^2+^ dynamics of isolated mouse beta-cells and islets: Implications for mathematical models. Biophys J.

[pbio-0040026-b005] De Vries G, Sherman A (2000). Channel sharing in pancreatic beta -cells revisited: enhancement of emergent bursting by noise. J Theor Biol.

[pbio-0040026-b006] Sherman A, Rinzel J, Keizer J (1988). Emergence of organized bursting in clusters of pancreatic beta-cells by channel sharing. Biophys J.

[pbio-0040026-b007] Kinard TA, de Vries G, Sherman A, Satin LS (1999). Modulation of the bursting properties of single mouse pancreatic beta-cells by artificial conductances. Biophys J.

[pbio-0040026-b008] Ravier MA, Guldenagel M, Charollais A, Gjinovci A, Caille D (2005). Loss of connexin36 channels alters beta-cell coupling, islet synchronization of glucose-induced Ca2+ and insulin oscillations, and basal insulin release. Diabetes.

[pbio-0040026-b009] Koster JC, Remedi MS, Flagg TP, Johnson JD, Markova KP (2002). Hyperinsulinism induced by targeted suppression of beta cell K_ATP_ channels. Proc Natl Acad Sci U S A.

[pbio-0040026-b010] Remedi M-S, Koster JC, Markova KP, Seino S, Miki T (2004). Diet-induced glucose intolerance in mice with decreased β-cell K_ATP_ channels. Diabetes.

[pbio-0040026-b011] Rocheleau JV, Walker GM, Head WS, McGuinness OP, Piston DW (2004). Microfluidic glucose stimulation reveals limited coordination of intracellular Ca^2+^ activity oscillations in pancreatic islets. Proc Natl Acad Sci U S A.

[pbio-0040026-b012] Shiota C, Larsson O, Shelton KD, Shiota M, Efanov AM (2002). Sulfonylurea receptor type 1 knock-out mice have intact feeding-stimulated insulin secretion despite marked impairment in their response to glucose. J Biol Chem.

[pbio-0040026-b013] Seghers V, Nakazaki M, DeMayo F, Aguilar-Bryan L, Bryan J (2000). Sur1 knockout mice. A model for K(ATP) channel-independent regulation of insulin secretion. J Biol Chem.

[pbio-0040026-b014] Miki T, Nagashima K, Tashiro F, Kotake K, Yoshitomi H (1998). Defective insulin secretion and enhanced insulin action in K_ATP_ channel-deficient mice. Proc Natl Acad Sci U S A.

[pbio-0040026-b015] Efendic S, Luft R, Grill V (1974). Effect of somatostatin on glucose induced insulin release in isolated perfused rat pancreas and isolated rat pancreatic islets. FEBS Lett.

[pbio-0040026-b016] Dufer M, Haspel D, Krippeit-Drews P, Aguilar-Bryan L, Bryan J (2004). Oscillations of membrane potential and cytosolic Ca(2+) concentration in SUR1(−/−) beta cells. Diabetologia.

[pbio-0040026-b017] Piston DW, Knobel SM, Postic C, Shelton KD, Magnuson MA (1999). Adenovirus-mediated knockout of a conditional glucokinase gene in isolated pancreatic islets reveals an essential role for proximal metabolic coupling events in glucose-stimulated insulin secretion. J Biol Chem.

[pbio-0040026-b018] Bennett BD, Jetton TL, Ying G, Magnuson MA, Piston DW (1996). Quantitative subcellular imaging of glucose metabolism within intact pancreatic islets. J Biol Chem.

[pbio-0040026-b019] Wollheim CB (2000). Beta-cell mitochondria in the regulation of insulin secretion: A new culprit in type II diabetes. Diabetologia.

[pbio-0040026-b020] Rizzo MA, Magnuson MA, Drain PF, Piston DW (2002). A functional link between glucokinase binding to insulin granules and conformational alterations in response to glucose and insulin. J Biol Chem.

[pbio-0040026-b021] Rizzo MA, Piston DW (2003). Regulation of beta cell glucokinase by S-nitrosylation and association with nitric oxide synthase. J Cell Biol.

[pbio-0040026-b022] Davidson JS, Baumgarten IM (1988). Glycyrrhetinic acid derivatives: A novel class of inhibitors of gap-junctional intercellular communication. Structure-activity relationships. J Pharmacol Exp Ther.

[pbio-0040026-b023] Cartier E, Conti LR, Vandenberg CA, Shyng S-L (2001). Defective trafficking and function of K_ATP_ channels caused by a sulfonylurea receptor 1 mutation associated with persistent hyperinsulinemic hypoglycemia of infancy. Proc Natl Acad Sci U S A.

[pbio-0040026-b024] Partridge CJ, Beech DJ, Sivaprasadarao A (2001). Identification and pharmacological correction of a membrane trafficking defect associated with a mutation in the sulfonylurea receptor causing familial hyperinsulinism. J Biol Chem.

[pbio-0040026-b025] Huopio H, Shyng SL, Otonkoski T, Nichols CG (2002). K(ATP) channels and insulin secretion disorders. Am J Physiol Endocrinol Metab.

[pbio-0040026-b026] Koster JC, Marshall BA, Ensor N, Corbett JA, Nichols CG (2000). Targeted overactivity of beta cell K(ATP) channels induces profound neonatal diabetes. Cell.

[pbio-0040026-b027] Gloyn AL, Pearson ER, Antcliff JF, Proks P, Bruining GJ (2004). Activating mutations in the gene encoding the ATP-sensitive potassium-channel subunit Kir6.2 and permanent neonatal diabetes. N Engl J Med.

[pbio-0040026-b028] Scharp DW, Kemp CB, Knight MJ, Ballinger WF, Lacy PE (1973). The use of ficoll in the preparation of viable islets of langerhans from the rat pancreas. Transplantation.

[pbio-0040026-b029] Stefan Y, Meda P, Neufeld M, Orci L (1987). Stimulation of insulin secretion reveals heterogeneity of pancreatic B cells in vivo. J Clin Invest.

[pbio-0040026-b030] McDonald JC, Duffy DC, Anderson JR, Chiu DT, Wu H (2000). Fabrication of microfluidic systems in poly(dimethylsiloxane). Electrophoresis.

[pbio-0040026-b031] Freedman JC, Novak TS (1989). Optical measurement of membrane potential in cells, organelles, and vesicles. Methods Enzymol.

